# Circulating anti‐glutamic acid decarboxylase‐65 antibody titers are positively associated with the capacity of insulin secretion in acute‐onset type 1 diabetes with short duration in a Japanese population

**DOI:** 10.1111/jdi.13052

**Published:** 2019-04-19

**Authors:** So Yamamura, Tomoyasu Fukui, Yusaku Mori, Toshiyuki Hayashi, Takeshi Yamamoto, Makoto Ohara, Ayako Fukase, Hiroto Sasamori, Tetsuro Kobayashi, Tsutomu Hirano

**Affiliations:** ^1^ Division of Diabetes Department of Medicine, Metabolism and Endocrinology Showa University School of Medicine Tokyo Japan; ^2^ Gaienhigashi Clinic Tokyo Japan; ^3^ Division of Immunology and Molecular Medicine Okinaka Memorial Institute for Medical Research Tokyo Japan

**Keywords:** C‐X‐C motif chemokine 10, Glutamic decarboxylase‐65, Type 1 diabetes

## Abstract

**Aims/Introduction:**

To elucidate the relationship between titers of islet autoantibodies, the C‐X‐C motif chemokine 10 – a circulating chemokine that activates T‐helper 1 cells leading to β‐cell destruction – and β‐cell function in type 1 diabetes.

**Materials and Methods:**

In total, 58 type 1 diabetes patients positive for glutamic decarboxylase‐65 autoantibodies (GADA)‐radioimmunoassay (mean age 54.1 years; 27 acute‐onset cases and 31 slowly progressive cases) were enrolled; serum C‐X‐C motif chemokine 10 (*n *=* *50), zinc transporter 8 autoantibodies (*n *=* *50) and GADA (*n *=* *58) by an enzyme‐linked immunosorbent assay, and insulinoma‐associated antigen‐2 autoantibodies by radioimmunoassay (*n *=* *50) were measured. The ratio of 100 × random C‐peptide (ng/mL)‐to‐plasma glucose levels (mg/dL; C‐peptide index [CPI]) was measured.

**Results:**

The CPI significantly decreased in both groups with the progression of disease duration. GADA titers by radioimmunoassay and enzyme‐linked immunosorbent assay were strongly correlated with the CPI in acute‐onset type 1 diabetes patients with a shorter disease duration (≤10 years), but not in those with a longer duration or slowly progressive type 1 diabetes. Neither insulinoma‐associated antigen‐2 nor zinc transporter 8 autoantibodies titers were correlated with the CPI. Serum C‐X‐C motif chemokine 10 levels in both groups were significantly higher than in non‐diabetic controls, and persisted at high levels even in those with chronic duration.

**Conclusions:**

Among islet autoantibodies, the intensity of the humoral immune response, as defined by GADA titers, reflected the degree of residual β‐cell function in acute‐onset type 1 diabetes patients with short duration. Prolonged disease activity might accelerate β‐cell impairment in both subtypes of type 1 diabetes.

## Graphical abstract

Among islet autoantibodies, the intensity of the humoral immune response, as measured by glutamic decarboxylase‐65 autoantibodies titers, reflected the degree of residual β‐cell function in acute‐onset type 1 diabetes patients with short duration.

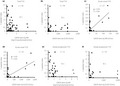

## Introduction

Type 1 diabetes is caused by autoimmune‐mediated selective destruction of insulin‐producing β‐cells^1^, ^2^. Although the precise etiology has not been elucidated, there is a general consensus that type 1 diabetes is mainly a T‐cell‐mediated disease triggered by environmental factors with subsequent defects in β‐cell antigen‐specific immune tolerance and destructive lymphocytic infiltration into islets producing C‐X‐C motif chemokine 10 (CXCL10). CXCL10 is an interferon‐γ‐induced chemokine that activates migrating T‐helper 1 (Th1) cells, and high circulating CXCL10 levels have been detected in newly diagnosed acute‐onset type 1 diabetes^3^ and fulminant type 1 diabetes^4^. In contrast, although unlikely to be intrinsically diabetogenic, several circulating islet autoantibodies against glutamic acid decarboxylase‐65 (GADA), insulinoma‐associated antigen protein 2 (IA‐2A) and insulin, which are generated from destroyed β‐cells, are strongly associated with the development of type 1 diabetes. It has been speculated that the appearance of autoantibodies to one or several islet autoantigens reflects the autoimmune pathogenesis of β‐cell killing. Among the several islet autoantibodies, GADA titers are measured worldwide by radioimmunoassay (RIA; GADA‐RIA), which is currently the gold standard, to identify individuals at high risk for type 1 diabetes^5^, and is frequently used to detect autoantibodies in patients with acute‐onset type 1 diabetes^6^.

Among the subtypes of type 1 diabetes, slowly progressive type 1 diabetes (otherwise known as latent autoimmune diabetes in adults) is a form of autoimmune diabetes defined by GADA‐RIA positivity, which is initially diagnosed as type 2 diabetes, as the subsequent gradual loss of β‐cell function progresses towards insulin dependency within several years after diagnosis^7^. Several studies have reported that higher GADA‐RIA titers are predictive of future complete β‐cell failure in slowly progressive type 1 diabetes^8^, ^9^, ^10^ and latent autoimmune diabetes in adults patients^11^, ^12^. Thus, high titers of GADA‐RIA seem to be associated future progressive β‐cell insufficiency in slowly progressive type 1 diabetes and latent autoimmune diabetes in adults patients. In contrast, few studies have evaluated the association between GADA‐RIA titers and disease activity in acute‐onset type 1 diabetes patients.

Recently, measurement of GADA has changed from RIA to non‐radioactive enzyme‐linked immunosorbent assay (GADA‐ELISA) in Japan. This commercially available GADA‐ELISA is reported to have higher specificity for the diagnosis of type 1 diabetes, as compared with GADA‐RIA^13^. However, the correlation between GADA titers, as measured with GADA‐ELISA, and residual β‐cell function has not been fully investigated in patients with type 1 diabetes.

Therefore, the present study was carried out in order to investigate the association among islet autoantibody status (type and number); that is, GADA by ELISA, IA‐2A by RIA and zinc transporter 8 autoantibodies (ZnT‐8A) by ELISA, and β‐cell function and serum CXCL10 levels in acute‐onset type 1 diabetes and slowly progressive type 1 diabetes patients positive for GADA by RIA.

## Methods

### Participants

A total of 58 patients with type 1 diabetes positive for GADA‐RIA were consecutively recruited from Showa University Hospital (Tokyo, Japan) in the present retrospective study, and the results were recently partly published^14^. GADA titers were measured simultaneously by the RIA and ELISA method in December 2015. IA‐2A and ZnT‐8A titers were also measured in 50 (86%) patients (*n *=* *27, acute‐onset type 1 diabetes; *n *=* *23, slowly progressive type 1 diabetes) using samples that had been stored at −80°C until analysis. These stored samples were taken in December 2015 when GADA‐RIA and GADA‐ELISA were measured simultaneously. Based on the diagnostic criteria and clinical information at diabetes onset, the patients were classified into two subtypes of type 1 diabetes: acute‐onset type 1 diabetes and slowly progressive type 1 diabetes^15^, ^16^. The non‐diabetic controls met the following criteria: (i) blood glucose levels ≤110 mg/dL in fasting or ≤140 mg/dL after eating; and (ii) glycated hemoglobin (HbA1c) value of <6.0%. The exclusion criteria were as follows: (i) age <20 years or >80 years; (ii) chronic hepatitis C; and (iii) evidence of chronic kidney disease (estimated glomerular filtration rate <60 mL/min/1.73 m^2^). In addition, the coexistence of autoimmune thyroid disease (AITD) was investigated using stored samples. AITD was defined as Graves' disease (the presence of thyroid‐stimulating hormone receptor antibodies) treated with anti‐thyroid drugs or Hashimoto thyroiditis (the presence of anti‐thyroid peroxidase antibody and/or thyroglobulin antibodies) treated with/without hormone replacement therapy.

The study protocol was approved by the ethics committee of the Showa University School of Medicine (permit number: 2612).

### Laboratory analysis

HbA1c content (%) was estimated as a National Glycohemoglobin Standardization Program‐equivalent value (%), as calculated by the following formula: HbA1c (%) = HbA1c (Japan Diabetes Society) (%) + 0.4%^17^. Serum C‐peptide and plasma glucose levels were measured with an immunoenzymometric assay and the glucose oxidase method, respectively.

### Islet autoantibody determination

Glutamic decarboxylase‐65 autoantibodies titers were measured by the RIA^18^ and ELISA^19^, with cut‐off values of 1.5 and 5.0 U/mL, respectively. Titers of IA‐2A and ZnT‐8A were measured by RIA^20^, with a cut‐off value of 0.4 U/mL (range 0.4–50 U/mL), and by ELISA^21^, with a cut‐off value of 15 U/mL (range 15–2000 U/mL), respectively. If the IA‐2A and ZnT‐8A titers fell below the lower limits of detection, a fill value of 0.01 was used. GADA titers, as assessed with an ELISA, were actual measures even if the value was <5.0 U/mL.

### Thyroid autoantibody determination

Thyroid‐stimulating hormone receptor antibodies, thyroid peroxidase antibody and thyroglobulin antibodies were determined by electrochemiluminescence immunoassay for anti‐thyroid‐stimulating hormone receptor, anti‐thyroid peroxidase and anti‐thyroglobulin (Roche Diagnostic GmbH, Mannheim, Germany), with cut‐off values of 2.0 U/L, 16.0 U/mL and 28.0 U/mL, respectively.

### Chemokine determination

Serum CXCL10 levels were measured using the Human CXCL10 ELISA kit (cat. no. 173194; Abcam, Cambridge, UK). The intra‐ and interassay variations of the CXCL10 ELISA were 5.1% and 11.1%, respectively.

### Assessment of insulin secretion ability

A ratio of 100 × random C‐peptide (ng/mL)‐to plasma glucose levels (mg/dL) (C‐peptide index [CPI]) was measured as the residual β‐cell function^22^, ^23^.

Of the 58 type 1 diabetes patients, the glucagon stimulation test (GST) was carried out in the morning (09.00 hours) after a 10‐h fast with 31 patients. The 6‐min value of C‐peptide immunoreactivity (CPR) after intravenous administration of 1 mg glucagon (stimulated‐CPR) was investigated.

### Statistical analysis

Comparisons between groups were carried out using the Steel–Dwass test, Mann–Whitney *U*‐test or χ^2^‐test. Non‐parametric correlations were identified using Spearman's rank correlation coefficient. Differences were considered significant at a two‐tailed probability (*P*) value of < 0.05. All statistical analyses were carried out with JMP Pro 13.0 software (SAS Institute Japan Ltd., Tokyo, Japan).

## Results

Patient characteristics are summarized in Table 1. The mean patient age and age at diabetes onset were significantly lower in the acute‐onset type 1 diabetes patients than in the slowly progressive type 1 diabetes patients. Mean height, bodyweight and body mass index were comparable between the subtypes of type 1 diabetes patients, but body mass index was significantly higher in slowly progressive type 1 diabetes patients than acute‐onset type 1 diabetes patients. As expected, serum C‐peptide levels and the CPI were significantly lower in the acute‐onset type 1 diabetes patients (both *P *<* *0.0001). HbA1c levels were comparable between the subtypes of type 1 diabetes patients. GADA‐RIA titers were higher in the slowly progressive type 1 diabetes patients than in the acute‐onset type 1 diabetes patients, whereas GADA‐ELISA titers were comparable between the subtypes of type 1 diabetes patients. Among the 58 GADA‐RIA‐positive type 1 diabetes patients, two (7.4%) of 27 patients with acute‐onset type 1 diabetes and 12 (38.7%) of 31 patients with slowly progressive type 1 diabetes were negative with the GADA‐ELISA.

**Table 1:** Patient characteristics

	Total	Acute‐onset T1D	Slowly progressive T1D	*P*‐value (AT1 vs SP1)
*n*	58	27	31	
Age (years)	53.5 ± 15.0	46.1 ± 13.2	60.0 ± 13.5	0.0002
Age at diabetes onset (years)	41.2 ± 15.8	34.4 ± 15.5	48.3 ± 13.0	0.0009
Duration of disease (years)	12.1 ± 9.3	12.6 ± 10.2	11.5 ± 8.6	NS
Height (cm)	161.2 ± 9.8	163.0 ± 9.0	159.0 ± 10.4	NS
Bodyweight (kg)	61.8 ± 13.1	59.8 ± 12.1	64.1 ± 13.9	NS
Body mass index (kg/m^2^)	23.5 ± 4.1	22.4 ± 3.5	24.8 ± 4.4	0.03
Glucose (mg/dL)	150.6 ± 50.1	152.93 ± 59.2	148.5 ± 41.3	NS
C‐peptide (ng/mL)*	0.75 ± 0.98	0.14 ± 0.25	1.29 ± 1.07	<0.0001
CPI	0.58 ± 0.83	0.11 ± 0.18	1.00 ± 0.95	<0.0001
HbA1c (%)	8.1 ± 1.7	8.1 ± 1.5	8.2 ± 1.8	NS
GADA‐RIA titers (IU/mL)	725.9 ± 3798.8	22.0 ± 39.2	1338.9 ± 5155.9	0.19
GADA‐ELISA titers (IU/mL)†	314.0 ± 582.2	339.6 ± 583.2	291.8 ± 590.2	NS
IA‐2A titers (AT1, *n* = 27; SP1, *n* = 23)‡	3.4 ± 8.7	6.1 ± 11.2	0.2 ± 0.5	0.0089
ZnT‐8A titers (AT1, *n* = 27; SP1, *n* = 23)§	31.2 ± 93.4	55.8 ± 122.6	1.7 ± 5.7	0.04
Total insulin dose/day (units)	30.4 ± 18.6	38.3 ± 17.7	23.6 ± 16.7	0.004
TDD/BW (units/kg)	0.5 ± 0.3	0.6 ± 0.3	0.4 ± 0.3	0.003
Thyroid disease, % (*n*)	30 (15/50)	26 (7/27)	35 (8/23)	NS

Values are presented as the mean ± standard deviation. * † ‡ §

AT1, acute‐onset type 1 diabetes; CPI, C‐peptide index; HbA1c, glycated hemoglobin; NS, not significant; RIA, radioimmunoassay; SP1, slowly‐progressive type 1 diabetes; T1D, type 1 diabetes; TDD/BW, total daily insulin dose/body weight.transporter‐8.

The mean titers of IA‐2A and ZnT‐8A were significantly higher in the acute‐onset type 1 diabetes patients than in the slowly progressive type 1 diabetes patients. The need for insulin was significantly greater for patients with acute‐onset type 1 diabetes than those with slowly progressive type 1 diabetes. The prevalence of thyroid disease was comparable between the subtypes of type 1 diabetes patients.

The prevalence of islet autoantibody in GADA‐RIA‐positive type 1 diabetes patients (*n *=* *50) is shown in Table S1. The prevalence the patients who were positive with the GADA‐RIA only was significantly higher in the slowly progressive type 1 diabetes group than in the acute‐onset type 1 diabetes group (83% vs 33%, respectively, *P *=* *0.0005), whereas the prevalence of multiple islet autoantibody‐positivity (IA‐2A, ZnT‐8A or both) was significantly lower (17% vs 67%, respectively, *P *=* *0.0005).

In addition, the prevalence of IA‐2A or ZnT‐8A in patients with acute‐onset type 1 diabetes was 59% (16 of 27) and 33% (9 of 27), respectively, which was significantly higher than the prevalence of those in patients with slowly progressive type 1 diabetes (13% and 9%, *P *=* *0.0008 and 0.036, respectively)

### GADA titers were positively correlated with residual β‐cell function in the acute‐onset type 1 diabetes

**Figure 1:**
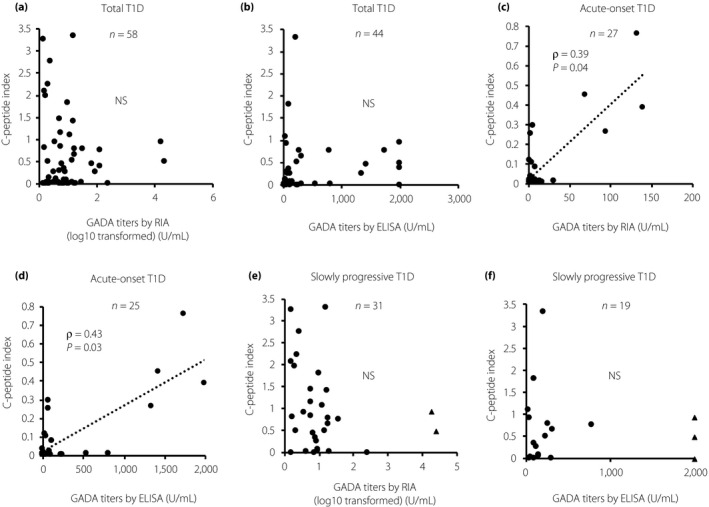
Correlation between autoantibodies against glutamic acid decarboxylase‐65 (GADA) titers and C‐peptide index (CPI; 100 × random C‐peptide (ng/mL) / plasma glucose levels [mg/dL]). (a,b) There was no correlation between GADA titers by radioimmunoassay (RIA; *n *=* *58) and enzyme‐linked immunosorbent assay (ELISA; *n *=* *44) and CPI among total type 1 diabetes patients. (c,d) GADA titers by RIA (*n *=* *27) and ELISA (*n *=* *25) were both significantly correlated with CPI in acute‐onset type 1 diabetes patients (*P *<* *0.05). (e,f) GADA titers by RIA (*n *=* *31) and ELISA (*n *=* *19) were not correlated with CPI in slowly progressive type 1 diabetes patients. NS, not significant; T1D, type 1 diabetes.

Figure 1 shows the correlation between GADA titers and the CPI in all type 1 diabetes patients (Figure 1a,b), the acute‐onset type 1 diabetes patients (Figure 1c,d) and the slowly progressive type 1 diabetes patients (Figure 1e,f). Correlation analysis was carried out only for patients positive for GADA‐RIA or GADA‐ELISA.

There was no correlation between GADA titers by RIA (*n *=* *58) and ELISA (*n *=* *44), and the CPI in all type 1 diabetes patients. However, GADA titers by RIA (*n *=* *27) and ELISA (*n *=* *25) were both significantly associated with the CPI in the acute‐onset type 1 diabetes patients (ρ = 0.39 and ρ = 0.43, *P *=* *0.04 and *P *=* *0.03, respectively). GADA titers by RIA (*n *=* *31) and ELISA (*n *=* *19) were not correlated to the CPI in the slowly progressive type 1 diabetes. As it is known that the coexistence of immune‐mediated encephalopathy^24^ or type 1 diabetes‐protective human leukocyte antigen^25^ are related to extremely high titers of GADA, we excluded slowly progressive type 1 diabetes patients (*n *=* *2 and *n *=* *3, closed triangle in Figure 1e,f, respectively) with extremely high titers of GADA (e.g., GADA‐RIA >10,000 IU/mL and GADA‐ELISA >2,000 IU/mL), and examined the correlation between GADA titers by RIA and ELISA, and the CPI in the SPIDDM patients. Neither GADA‐RIA nor GADA‐ELISA titers were associated with the CPI in these patients.

Next, we analyzed the correlation between GADA titers by RIA and ELISA, and the CPI between the acute‐onset type 1 diabetes patients with shorter disease duration versus those with a longer disease duration (≤10 vs >10 years, respectively). As shown in Figure S1, there was a significant correlation between GADA titers by RIA and ELISA, and the CPI in those with a shorter duration of diabetes (ρ = 0.85 and ρ = 0.83, *P *<* *0.0001 and *P *=* *0.0002, respectively), but not in those with a longer duration of diabetes. For assessing subsequent β‐cell function in acute‐onset type 1 diabetes patients with a shorter duration of diabetes with relatively preserved the CPI (*n *=* *4, open circles in Figure S1a,b), the changes in the CPI after 2 years were also investigated among these patients. Thereafter, no significant decline in the CPI was observed (0.47 ± 0.21 vs 0.42 ± 0.53).

Neither IA‐2A nor ZnT‐8A titers showed an association with the CPI in all patients with type 1 diabetes, acute‐onset type 1 diabetes or slowly progressive type 1 diabetes irrespective of disease duration (data not shown).

### Association GADA titers and the CPI in patients with type 1 diabetes with/without autoimmune thyroiditis

As the coexistence of AITD is associated with high GADA titers^26^, we assessed the GADA titers and correlation between GADA titers and the CPI in type 1 diabetes patients with AITD and those without AITD.

**Figure 2:**
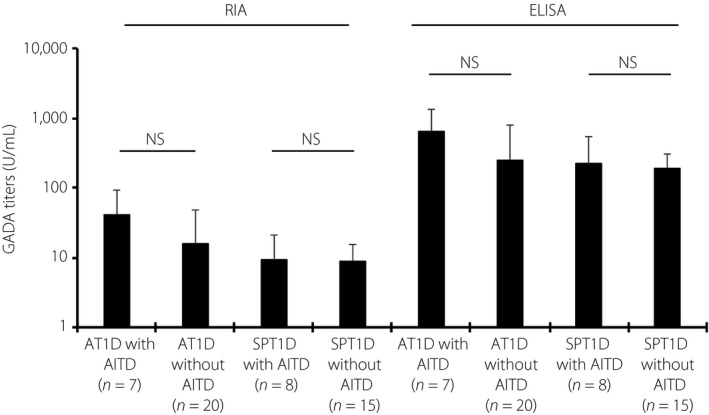
Autoantibodies against glutamic acid decarboxylase‐65 (GADA) titers in patients with acute‐onset type 1 diabetes (T1D) and slowly progressive type 1 diabetes with/without autoimmune thyroiditis (AITD). GADA titers by radioimmunoassay (RIA) and enzyme‐linked immunosorbent assay (ELISA) were comparable in both subtypes of type 1 diabetes with/without AITD. AT1D, acute‐onset type 1 diabetes; SPT1D, slowly progressive type 1 diabetes; T1D, type 1 diabetes.

Unexpectedly, GADA titers by RIA and ELISA were comparable in both subtypes of type 1 diabetes patients with/without AITD (Figure 2). GADA titers by RIA and ELISA were positively associated with the CPI in patients with acute‐onset type 1 diabetes with AITD (*n *=* *7, both ρ = 0.79, *P *<* *0.05), but not in those without AITD (*n *=* *20). In contrast, GADA titers, not ELISA, by RIA were negatively associated with the CPI in patients with slowly progressive type 1 diabetes without AITD (*n *=* *15), but not in those with AITD (Figure S2).

### Change in residual β‐cell function and GADA titers over time

**Figure 3:**
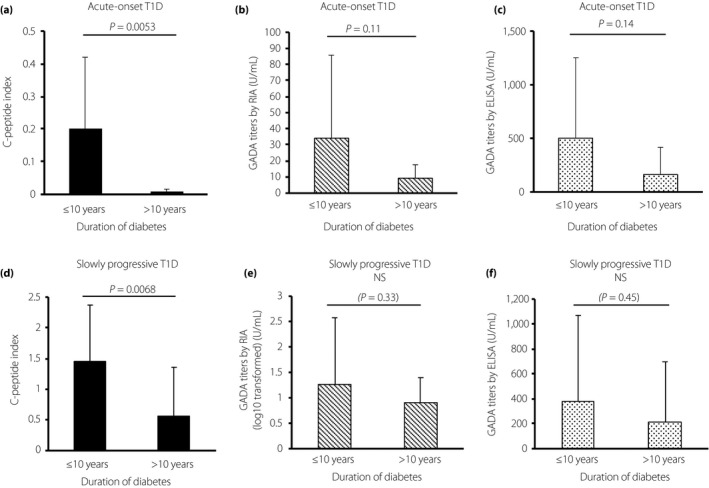
Change in residual β‐cell function and autoantibodies against glutamic acid decarboxylase‐65 (GADA) titers over time. C‐peptide index (CPI; 100 × random C‐peptide [ng/mL] / plasma glucose levels [mg/dL]) significantly decreased in (a) acute‐onset type 1 diabetes and (d) slowly progressive type 1 diabetes. GADA titers by radioimmunoassay (RIA) and enzyme‐linked immunosorbent assay (ELISA) tended to (b,c) decrease in acute‐onset type 1 diabetes, (e,f) but were almost unchanged in slowly progressive type 1 diabetes. Error bars represent standard deviation. NS, not significant; T1D, type 1 diabetes.

We investigated the change in the CPI and GADA titers by RIA and ELISA between patients with a shorter duration of diabetes versus a longer duration of diabetes (≤10 versus >10 years, respectively). The results showed that with the progression of disease duration, the CPI significantly declined in both subtypes of type 1 diabetes (Figure 3a,d), whereas GADA titers by RIA and ELISA tended to decrease in the acute‐onset type 1 diabetes (Figure 3b,c), but was almost unchanged in the slowly progressive type 1 diabetes (Figure 3e,f). A duration‐dependent decline in the IA‐2A and ZnT‐8A titers was also observed (≤10 vs >10 years, *P *=* *0.07 and *P *=* *0.043, respectively).

### Correlation between multiple islet autoantibodies and clinical parameters

Next, we investigated whether positivity for multiple islet autoantibodies (IA‐2A, ZnT‐8A or both) influenced the CPI, glycemic control or age at diabetes onset among GADA‐RIA‐positive type 1 diabetes patients (*n *=* *50). The patients who tested positive for IA‐2A or ZnT‐8A, or both, had a significantly lower CPI than those positive for GADA‐RIA only. HbA1c levels and age at diabetes onset were similar among the three groups (Figure S3).

In addition, we investigated the role of IA‐2A or ZnT‐8A positivity on the CPI among the IA‐2A‐positive patients (19/50, 38%) versus IA‐2A‐negative patients (31/50, 62%) and ZnT‐8A‐positive patients (11/50, 22%) versus ZnT‐8A‐negative patients (39/50, 78%). As it turned out, patients who were positive for IA‐2A had a significantly lower CPI than those negative for IA‐2A (0.24 ± 0.38 vs 0.75 ± 0.92, *P *=* *0.03, respectively), but there was no significant difference between ZnT‐8A‐positive versus ZnT‐8A‐negative patients (Table S2).

### Correlation between serum CXCL10 levels and clinical parameters

**Figure 4:**
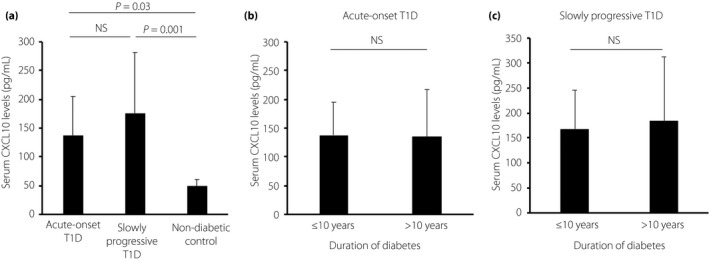
Serum C‐X‐C motif chemokine 10 (CXCL10) levels in patients with type 1 diabetes. Serum CXCL10 levels were (a) significantly higher in patients with acute‐onset type 1 diabetes (*n *=* *27) or slowly progressive type 1 diabetes (*n *=* *23) than in non‐diabetic controls, (b,c) and persisted in high levels even in those with chronic duration. Error bars represent standard deviation. NS, not significant; T1D, type 1 diabetes.

Serum CXCL10 levels were significantly higher in patients with acute‐onset type 1 diabetes (*n *=* *27) or slowly progressive type 1 diabetes (*n *=* *23) than in non‐diabetic controls (*n *=* *8; age 35 ± 12 years; Figure 4a). Serum CXCL10 levels persisted at high levels, even in those with chronic duration (Figure 4b,c). As serum CXCL10 levels are increased in AITD patients^27^, ^28^, we assessed whether the co‐occurrence of AITD could augment serum CXCL10 levels in both subtypes of type 1 diabetes patients. Serum CXCL‐10 levels were significantly higher in patients with acute‐onset type 1 diabetes or slowly progressive type 1 diabetes, irrespective of the co‐occurrence of AITD, than in non‐diabetic controls (acute‐onset type 1 diabetes with AITD, *n *=* *7: 166.7 ± 81.6 pg/mL; acute‐onset type 1 diabetes without AITD, *n *=* *20: 125.9 ± 62.5 pg/mL; slowly progressive type 1 diabetes with AITD, *n *=* *8: 206.3 ± 140.0 pg/mL; slowly progressive type 1 diabetes without AITD, *n *=* *15: 160.2 ± 83.5 pg/mL vs non‐diabetic controls: 49.1 ± 11.5 pg/mL, all *P *<* *0.001).

In addition, there was no significant difference in serum CXCL10 levels in both subtypes of type 1 diabetes patients irrespective of the co‐occurrence of AITD, when classified according to duration of diabetes (≤10 vs >10 years; data not shown).

No correlation was observed between serum CXCL10 levels and CPI, age, duration of diabetes and titers of islet autoantibodies in both subtypes of type 1 diabetes patients. Among type 1 diabetes patients, a multiple islet autoantibody was not associated with further enhanced serum CXCL‐10 levels (data not shown).

### CPI was significantly associated with stimulated‐CPR by GST

Since stimulated‐CPR by GST is widely accepted as a measure of β‐cell function^29^, ^30^, we assessed the correlation between the CPI assessed as β‐cell function in the present study and stimulated‐CPR by GST. The CPI was significantly correlated with stimulated‐CPR by GST in all type 1 diabetes, acute‐onset type 1 diabetes and slowly progressive type 1 diabetes patients (*n *=* *31, ρ = 0.98, *P *<* *0.001; *n *=* *10, ρ = 0.68, *P *=* *0.02; *n *=* *21, ρ = 0.93, *P *<* *0.001, respectively, Figure S4).

## Discussion

The results of the current study clearly showed that GADA titers by RIA and ELISA were positively correlated with the CPI in patients with acute‐onset type 1 diabetes, especially those with a disease duration of ≤10 years, suggesting that the intensity of the humoral immune response against GAD65 might reflect the degree of residual β‐cell function.

A study by Harrison *et al*.^31^ investigating the correlation between GADA titers and reactivity of T cells to GAD antigens among first relatives of type 1 diabetes patients showed that higher GADA titers (Th2‐dominant state) were correlated with a lower response of T cells against the GAD antigen (weaker Th1 response state), indicating that higher GADA titers were correlated with a slower disease progression. In an animal model, type 1 diabetes developed when there was an imbalance in the immune system from a Th2‐ to a Th1‐dominant state. Gabbay *et al*.^32^ also reported that GADA titers were inversely correlated with serum CXCL10 levels in adolescent patients with newly diagnosed type 1 diabetes. However, in the present study, there was no correlation between GADA titers and serum CXCL10 levels in the acute‐onset type 1 diabetes patients with a shorter duration of diabetes. In comparison with previous studies^32^, the participants in the present study were older at diabetes onset, had a longer disease duration and might have had different ancestries, which could explain the lack of a negative correlation between serum CXCL10 levels and GADA titers. Interestingly, high serum CXCL10 levels were almost unchanged despite progression of disease duration in both subtypes of type 1 diabetes patients (Figure 3). Shimada *et al*. reported that serum CXCL10 levels are significantly associated with the number of GAD65‐reactive T cells in the periphery of type 1 diabetes patients^33^. Accordingly, prolonged Th1 activity might accelerate functional β‐cell impairment in both subtypes of type 1 diabetes patients, although the current study could not show a negative correlation between serum CXCL10 levels and the CPI. As CXCL10 and its receptor, CXCR3, play a critical role in the autoimmune process of β‐cell exhaustion in type 1 diabetes patients^3^, ^33^, blocking CXCL10 seems to be a possible approach to alter the disease course of type 1 diabetes, even in the chronic stage.

Among slowly progressive type 1 diabetes patients, there was no correlation between GADA titers by RIA and ELISA, and the CPI. Interestingly, GADA‐RIA titers, not GADA‐ELISA, were negatively associated with the CPI among patients with slowly progressive type 1 diabetes without AITD (*n *=* *15), but not in those with AITD (Figure S2). However, as the mean titers of GADA‐RIA in SPIDDM patients with AITD are as low as 9.3 IU/mL, ranging from 1.5 to 35.6 IU/mL, which is extremely low compared with that in typical SPIDDM patients^8^, ^9^, ^10^, its interpretation would need to be carefully considered. Several studies have reported that high GADA‐RIA titers (≥10 U/mL) are associated with the progression of β‐cell failure and are also predictive of future insulin requirements in slowly progressive type 1 diabetes patients^8^. However, in the present study, there were no remarkable differences in the CPI between the two groups classified according to GADA‐RIA titers (≥10 vs <10 U/mL: 0.98 ± 0.90 vs 1.01 ± 1.01, *P *=* *0.94, respectively). These results show that prospective studies might be better to show the relationship between GADA‐RIA titers and residual β‐cell function in slowly progressive type 1 diabetes patients rather than cross‐sectional studies^8^, ^9^. In the present study, 12 (38.7%) of 31 patients with slowly progressive type 1 diabetes reverted to seronegativity on GADA‐ELISA, showing higher frequency versus acute‐onset type 1 diabetes (7%, *P *<* *0.0001), in agreement with previous reports^13^, ^34^. Although the reason for this seroconversion, especially in slowly progressive type 1 diabetes patients, remains to be elucidated, the CPI of those who became negative by GADA‐ELISA was significantly preserved as compared with those who were positive (1.57 ± 0.90 vs 0.66 ± 0.85, *P *=* *0.0008). This finding indicates that the disease of patients negative by GADA‐ELISA would run a less progressive course than that of those who were positive. The GADA‐ELISA appears to detect high‐affinity antibodies while ignoring low‐affinity antibodies less associated with progression to β‐cell exhaustion among slowly progressive type 1 diabetes patients^35^.

It is now well established that the rate of disease progression is considerably increased in non‐diabetic individuals with multiple islet autoantibodies than those with single autoantibodies^36^, ^37^, ^38^. In addition, measurement of a combination of other islet autoantibodies has been suggested as a useful diagnostic tool to identify autoimmune type 1 diabetes. Acute‐onset type 1 diabetes patients had a significantly higher prevalence of autoantibodies to IA‐2A or ZnT‐8A, as compared with slowly progressive type 1 diabetes patients, suggesting that the autoimmune process of β‐cell destruction is deeply involved in acute‐onset type 1 diabetes rather than slowly progressive type 1 diabetes. IA‐2A is known as an autoantibody reflective of the severity of β‐cell destruction more so than GADA^39^, but the correlation between ZnT‐8A and the severity of β‐cell function is not fully understood. The patients positive for IA‐2A had a significantly lower CPI than those negative for IA‐2A, but there was no significant difference between ZnT‐8A‐positive versus ZnT‐8A‐negative patients (Table S2). These results suggest that the presence of IA‐2A reflects more aggressive β‐cell destruction, as compared with that of ZnT‐8A, among patients with type 1 diabetes positive for GADA‐RIA.

The hallmark of type 1 diabetes is the deterioration of endogenous insulin secretion. Indeed, the CPI was remarkably decreased closer to complete exhaustion of β‐cell function in the acute‐onset type 1 diabetes patients with a longer disease duration (Figure 2a). Regarding procedures for determination of β‐cell function, a strong correlation between the CPI and stimulated CPR obtained by GST was observed (Figure S4). Thus, it could be considered that the CPI reflects β‐cell function equal to the results of GST, and is valid for the determination of β‐cell function. In agreement with the present study, Meier *et al*. reported a close correlation between a C‐peptide‐to‐glucose ratio after oral glucose ingestion and β‐cell area in humans^40^.

There were several limitations to the present study that should be addressed. First, the current study used a relatively small sample and the retrospective cross‐sectional design limited the precision of the results. However, we believe that current study is informative considering that few studies have investigated the association among islet autoantibody status, β‐cell function and serum CXCL10 levels in patients with acute‐onset type 1 diabetes or slowly progressive type 1 diabetes. Second, we did not collect the genetic markers (i.e., human leucocyte antigen, cytotoxic T lymphocyte antigen 4 and protein tyrosine phosphatase, non‐receptor 22 gene), the important genetic regulating elements of the development and progression of type 1 diabetes. Third, type 1 diabetes is characterized by T cells mediated β‐cell destruction^1^, ^2^. Regulatory T cells have been shown to be defective in this setting. As recent research has shown that the pathogenesis of type 1 diabetes is mainly caused by the disequilibrium between effector T cells and regulatory T cells^41^, ^42^, research using T cell assays would be required to assess the influence of disease activity, as defined by the numbers of islet antigen‐specific effector T cells and regulatory T cells, on the islet autoantibody status and β‐cell function in patients with type 1 diabetes in the future.

In conclusion, GADA titers by RIA and ELISA reflect the degree of residual β‐cell function in acute‐onset type 1 diabetes with short duration. The persistence of high serum CXCL10 levels indicates that prolonged disease activity might accelerate functional β‐cell impairment in patients with acute‐onset type 1 diabetes or slowly progressive type 1 diabetes.

This research received no specific grant from any funding agency in the public, commercial or not‐for‐profit sectors.

## Disclosure

The authors declare no conflict of interest.

## Supporting information


**Figure S1 ¦** Correlation between autoantibodies against glutamic acid decarboxylase‐65 (GADA) titers by radioimmunoassay (RIA) and enzyme‐linked immunosorbent assay (ELISA) and C‐peptide index (CPI; 100 × random C‐peptide [ng/mL] / plasma glucose levels [mg/dL]) among patients with acute‐onset type 1 diabetes with shorter duration of diabetes (≤10 years) and longer duration of diabetes (>10 years). (a,b) A strong significant correlation between GADA titers by RIA and ELISA, and CPI was observed in patients with acute‐onset type 1 diabetes with short duration of diabetes (both for *P *<* *0.0001; *n *=* *4, open circles: those with relatively preserved CPI), (c,d) but not in those with long duration of diabetes. T1D, type 1 diabetes.

**Figure S2 ¦** Correlation between autoantibodies against glutamic acid decarboxylase‐65 (GADA) titers and C‐peptide index (CPI; 100 × random C‐peptide (ng/mL) / plasma glucose levels [mg/dL]) in subtypes of type 1 diabetes patients with/without autoimmune thyroiditis (AITD).

**Figure S3 ¦** Association between number of islet autoantibodies positivity and clinical parameters.

**Figure S4 ¦** Correlation between 6‐min value of C‐peptide immunoreactivity (CPR) after intravenous administration of 1 mg glucagon (stimulated‐CPR) by glucagon stimulating test (GST) and C‐peptide index (CPI; 100 × random C‐peptide (ng/mL) / plasma glucose levels [mg/dL]).

**Table S1 ¦** Prevalence of glutamic decarboxylase‐65 autoantibodies, insulinoma‐associated antigen‐2 and zinc transporter 8 autoantibodies in acute‐onset and slowly progressive type 1 diabetes patients.

**Table S2 ¦** C‐peptide index among the insulinoma‐associated antigen‐2‐positive patients versus insulinoma‐associated antigen‐2‐negative patients and zinc transporter 8 autoantibodies‐positive patients versus zinc transporter 8 autoantibodies‐negative patients.

## Footnotes

* When C‐peptide levels fell below the lower limit of detection (i.e., <0.02), a fill value of 0.001 was used.

† Autoantibodies to glutamic acid decarboxylase‐65 (GADA) titers, as assessed with an enzyme‐linked immunosorbent assay (ELISA), were actual measured values even when <5.0 U/mL.

‡ When antibodies to an insulinoma‐associated protein 2 (IA‐2A) titers fell below the lower limit of detection (i.e., <0.4), a fill value of 0.01 was used.

§ When antibodies to zinc transporter‐8 (ZnT‐8A) titers fell below the lower limit of detection (i.e., <0.4), a fill value of 0.01 was used.
