# Supplementary material for: Circulating anti‐glutamic acid decarboxylase‐65 antibody titers are positively associated with the capacity of insulin secretion in acute‐onset type 1 diabetes with short duration in a Japanese population
Source: J Diabetes Investig. 2019 Apr 19;10(6):1480–9. doi: 10.1111/jdi.13052 (PMC7663970; doi:10.1111/jdi.13052)
Supplement: Supplementary file 5 — Table S1 ¦ Prevalence of glutamic decarboxylase‐65 autoantibodies, insulinoma‐associated antigen‐2 and zinc transporter 8 autoantibodies in acute‐onset and slowly progressive type 1 diabetes patients. [file JDI-10-1480-s005.docx]

Supplementary Table 1. Prevalence of autoantibodies against GAD, IA-2, and ZnT-8 among GADA-RIA-positive acute-onset and slowly progressive T1D patients

|  | **Total** | **Acute-onset**  **T1D** | **Slowly progressive**  **T1D** | ***P* value**  **(AT1 *vs*. SP1)** |
| --- | --- | --- | --- | --- |
|  | *n* = 50 | *n* = 27 | *n* = 23 |  |
| GADA-RIA alone | 28/50 (56%) | 9/27 (33%) | 19/23 (83%) | 0.0005 |
| ≥2 autoantibodies* | 24/50 (48%) | 18/27 (67%) | 4/23 (17%) | 0.0005 |
| 3 autoantibodies | 7/50 (14%) | 7/27 (24%) | 0/23 (0%) | 0.0085 |
| IA-2A and GADA-RIA | 19/50 (38%) | 16/27 (59%) | 3/23 (13%) | 0.0008 |
| ZnT-8A and GADA-RIA | 11/50 (22%) | 9/27 (33%) | 2/23 (9%) | 0.0361 |

* Positive for IA-2A, ZnT8A, or both.

Abbreviations: AT1, acute-onset type 1 diabetes; GADA, autoantibodies to glutamic acid decarboxylase-65; IA-2A, autoantibodies to insulinoma-associated protein 2; RIA, radioimmunoassay; SP1, slowly-progressive type 1 diabetes; T1D, type 1 diabetes; ZnT-8A, autoantibodies to zinc transporter-8
