# Supplementary material for: Circulating anti‐glutamic acid decarboxylase‐65 antibody titers are positively associated with the capacity of insulin secretion in acute‐onset type 1 diabetes with short duration in a Japanese population
Source: J Diabetes Investig. 2019 Apr 19;10(6):1480–9. doi: 10.1111/jdi.13052 (PMC7663970; doi:10.1111/jdi.13052)
Supplement: Supplementary file 6 — Table S2 ¦ C‐peptide index among the insulinoma‐associated antigen‐2‐positive patients versus insulinoma‐associated antigen‐2‐negative patients and zinc transporter 8 autoantibodies‐positive patients versus zinc transporter 8 autoantibodies‐negative patients. [file JDI-10-1480-s006.docx]

Supplementary Table 2. CPI among the IA-2A-positive patients vs. -negative patients and ZnT-8A-positive patients vs. -negative patients

|  | **Total** | **IA-2A-positive**  **+ GADA by RIA-positive T1D** | **IA-2A-negative**  **+ GADA by RIA-positive**  **T1D** | ***P* value** |
| --- | --- | --- | --- | --- |
|  | *n* = 50 | *n* = 19 | *n* = 31 |  |
| CPI | 0.56 ± 0.79 | 0.24 ± 0.38 | 0.75 ± 0.92 | 0.0262 |

|  | **Total** | **ZnT-8A-positive**  **+ GADA by RIA-positive T1D** | **ZnT-8A-negative**  **+ GADA by RIA-positive**  **T1D** | ***P* value** |
| --- | --- | --- | --- | --- |
|  | *n* = 50 | *n* = 11 | *n* = 39 |  |
| CPI | 0.56 ± 0.79 | 0.28 ± 0.31 | 0.63 ± 0.87 | 0.1665 |

Abbreviations: CPI: C-peptide index (100×random C-peptide (ng/mL)/plasma glucose levels (mg/dL)); IA-2A, autoantibodies to insulinoma-associated protein 2; GADA, autoantibodies to glutamic acid decarboxylase-65; RIA, radioimmunoassay; T1D, type 1 diabetes; Zn-T8A, autoantibodies to zinc transporter-8
